# Inhibitory Effect and Mechanism of the Down-Regulation of TRIM32 in Colorectal Cancer

**DOI:** 10.3390/ijms26115047

**Published:** 2025-05-23

**Authors:** Jiayu Ning, Xiaohua Cai, Yintong Su, Xingxing Fan, Mei Shen

**Affiliations:** Guangdong Provincial Key Laboratory of Tropical Disease Research, Department of Hygiene Inspection & Quarantine Science, School of Public Health, Southern Medical University, Guangzhou 510515, China; ningjiayu202@163.com (J.N.); 13421770036@163.com (X.C.); suesuyt@163.com (Y.S.); 13424081929@163.com (X.F.)

**Keywords:** TRIM32, colorectal cancer, prognosis, proliferation, migration

## Abstract

TRIM32 protein represents a crucial member of TRIM family that is highly expressed in numerous human cancers, and is associated with a poor prognosis. However, the mechanism of TRIM32 in colorectal cancer (CRC) is unclear. The expression of TRIM32 and its prognostic value in CRC were analyzed using The Cancer Genome Atlas (TCGA) and Gene Expression Omnibus (GEO) database. Real-time quantitative PCR, immunohistochemistry (IHC), and cell proliferation assays were used to explore the effects of down-regulation of TRIM32 expression on the proliferation, migration, and apoptosis of cultured CRC cells (HCT116 and SW480 cells) and in xenogeneic tumorigenic animals. Bioinformatics analysis showed that TRIM32 is up-regulated in many types of cancers, and exhibits significant prognostic value in CRC. Western blotting results showed that after knocking down TRIM32, the expression level of IκBα increased, and the expression levels of TRIM32, p-p65, Bcl-2, and IKKβ decreased. The inhibitory effect of TRIM32 on CRC in vivo was evaluated by measuring tumor volume and weight, Hematoxylin and eosin (H&E) staining, and Ki67 IHC staining in heterotopic tumor-forming mice with CRC. Down-regulation of TRIM32 can inhibit the activation of the NF-κB signaling pathway and the occurrence of CRC. Our research provides a new insight into the pathogenesis of CRC, and a therapeutic target for the treatment of CRC.

## 1. Introduction

Colorectal cancer (CRC) ranks as the third most prevalent cancer in the world, and the second leading cause of cancer-related death [1]. The GLOBOCAN 2020 database reveals that the worldwide incidence of CRC is increasing [2], and it is projected that by 2040 the number of new CRC cases will soar to 3.2 million [3]. Thus, it is important to develop novel and effective treatments for CRC.

Tripartite motif (TRIM) proteins, among one of the largest protein families in mammals, possess E3 ubiquitin ligase activity. Their *N*-terminal typically comprises a RING domain, two B-boxes (B1 and B2), and a coiled-coil (CC) region [4], with each domain having an independent function. Specifically, the TRIM32 protein, in addition to these common *N*-terminal domains, also harbors 6 NHL domains with repetitive sequences. TRIM proteins are engaged in a broad spectrum of biological processes and have various functions in cellular activities, including intracellular signal transduction, development, apoptosis, protein quality control, innate immunity, autophagy, and carcinogenesis [5]. Many studies have shown that members of the TRIM family are involved in tumorigenesis and cancer development, and TRIM proteins can be under-expressed or over-expressed in different cancers, such as prostate cancer, squamous cell carcinoma, and lung cancer [6,7,8].

The RING domain of TRIM32 endows it with E3 ubiquitin ligase activity, enabling it to regulate diverse biological processes such as apoptosis, transcription, the cell cycle, and tyrosine kinase growth factor receptor signal transduction [9]. In the context of the TRIM protein family, some members have been reported to exhibit cancer-inhibitory functions under certain physiological conditions. However, TRIM32 presents a distinct pattern. Multiple studies [10,11,12,13] have demonstrated that the expression of TRIM32 is significantly up-regulated in breast cancer, gastric cancer, pancreatic cancer, and lung cancer. In gastric cancer (GC) cells, TRIM32 functions as a proliferation and anti-apoptosis factor. It participates in the AKT pathway of GC cells, mediates glycolytic metabolism by targeting GLUT1 and HKII in GC cells, and significantly promotes the tumorigenicity of GC cells in vivo [14]. TRIM32 can maintain mitochondrial membrane potential after cisplatin treatment and reduce the generation of reactive oxygen species (ROS), reduce cisplatin sensitivity, and maintain Mitochondrial Membrane Potential Difference (MMPD) in non-small cell lung cancer (NSCLC) cells. TRIM32 overexpression down-regulates caspase 3 cleavage and cytochrome C release, and also positively regulates Bcl-2 protein expression and NF-κB signal transduction [15]. TRIM32 is up-regulated in benign and malignant tumors, and plays a carcinogenic role in tumor growth.

Studies have shown that the carcinogenesis of TRIM32 is related to its promotion of ubiquitination and degradation of Abi2, which impacts cell growth, migration, and transformation, and the expression of TRIM32 is elevated in human head and neck squamous cell carcinoma [16]. In addition, TRIM32 increases the radio-resistance of triple-negative breast cancer by promoting STAT3 transcriptional activation, and high expression is associated with a poor prognosis [17]. In the realm of colorectal cancer (CRC), some previous studies have preliminarily explored the expression of TRIM32 in CRC tissues. However, these studies are quite limited. They mainly concentrated on detecting the expression level, without in-depth exploration of the mechanism and biological function of TRIM32 in the occurrence and development of CRC. Therefore, the in-depth mechanism and biological function of TRIM32 in CRC still remain obscure. This study aims to bridge this gap and provide new insights into the role of TRIM32 in CRC.

Current research has shown that there is a close correlation between various proteins and the Nuclear factor-kappa B (NF-κB) signaling pathway. In the TRIM family, the interaction mechanisms between some members and the NF-κB pathway have been relatively well-defined. TRIM22 can enhance the K63-linked ubiquitination of IKKγ in glioblastoma, effectively activating the NF-κB signaling pathway and triggering tumor cell proliferation and tumor formation [18]. In addition, a large number of studies have demonstrated that E3 ubiquitin ligases play an important role in regulating the NF-κB pathway during tumorigenesis and development [19]. In colorectal cancer research, ABLIM1, as a novel E3 ligase, can specifically bind to IĸBα and promote its ubiquitination and degradation, activating the NF-κB/CCL-20 signaling pathway and significantly promoting the malignant biological behaviors of CRC cells. These studies fully demonstrate that various E3 ubiquitin ligases can change the activity of the NF-κB pathway by regulating the ubiquitination and degradation of related proteins in the NF-κB pathway, and are deeply involved in the tumorigenic process [20]. Nuclear factor-kappa B (NF-κB) transcription factor is the most crucial nuclear transcription factor, and exhibits multi-directional transcription regulation during intracellular signal transmission. It is a heterodimeric protein composed of two protein subunits, p65 and p50, which are the key to the activation and nuclear translocation of NF-κB [21]. NF-κB is a stress-responsive factor in the cellular environment, and participates in various biological processes including the immune response, inflammation, and cell growth, survival, and development [22]. The NF-κB signaling pathway has an important regulatory role in the occurrence and development of CRC.

We hypothesize that the down-regulation of TRIM32 can inhibit the activation of the NF-κB signaling pathway, thus affecting the progress of CRC. In this study, The Cancer Genome Atlas (TCGA) and Gene Expression Omnibus (GEO) database were utilized to analyze the expression of TRIM32. The specific effects of down-regulation of TRIM32 expression on the proliferation, migration, and apoptosis of CRC cells were investigated, and the anti-tumor effect and molecular mechanism of TRIM32 on CRC were examined by Western blotting. An in vivo model was used to verify the inhibitory effect of TRIM32 on CRC using tumor volume and weight measurement, Hematoxylin and eosin (H&E) staining, and Ki67 immunohistochemistry (IHC) staining of tumor tissue.

## 2. Results

### 2.1. TRIM32 Expression Is Up-Regulated in Human CRC Tissues

**Figure 1:**
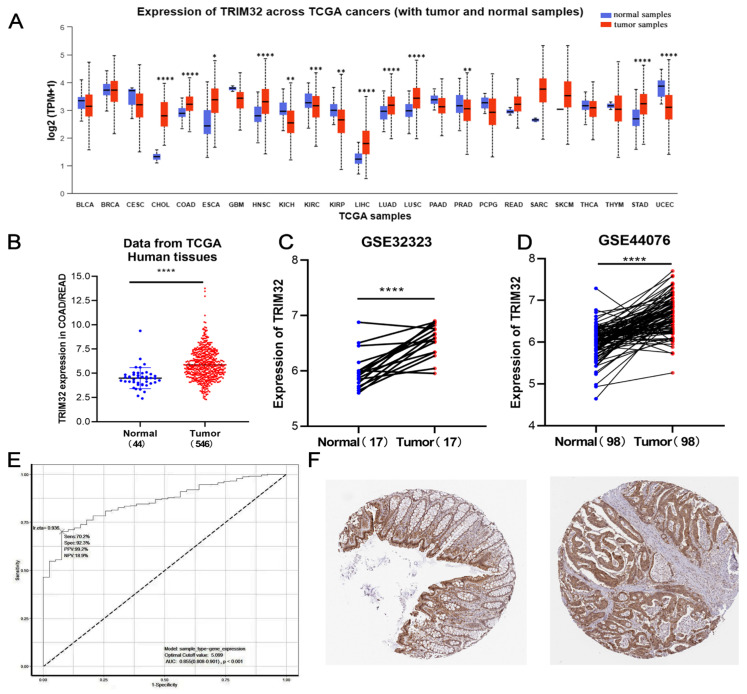
Relative expression of TRIM32 in pan-cancer. (**A**) Analysis of the difference in TRIM32 mRNA expression between COAD/READ patients’ cancer tissues and adjacent normal tissues using TCGA database. (**B**) Expression of TRIM32 in normal (*n* = 44) and tumor (*n* = 566) tissues from TCGA database. (**C**,**D**) Analysis of TRIM32 expression based on GSE32323 (*n* = 17) and GSE44076 (*n* = 98) datasets. (**E**) ROC curve analysis of the diagnostic value of TRIM32 for CRC. (**F**) Immunohistochemical analysis of TRIM32 expression in normal colon tissues and colon cancer tissues from HPA database (* *p* < 0.05, ** *p* < 0.01, *** *p* < 0.001,**** *p* < 0.001).

The expression of TRIM32 in human tumor tissues was initially analyzed using the UALCAN database (http://ualcan.path.uab.edu/ (accessed on 1 January 2023)). The results showed that TRIM32 is up-regulated in eight tumor tissues, namely, CHOL, COAD, ESCA, HNSC, LIHC, LUAD, LUSC, and STAD. CRC-related data in TCGA and GEO were examined, and the results showed that TRIM32 expression was significantly up-regulated in CRC tissues (*p* < 0.001) (Figure 1A–D). The IHC results of the TRIM32 protein in the HPA database showed that TRIM32 was expressed in the nuclei of normal tissues and cancerous tissues, and the expression of TRIM32 was notably up-regulated in CRC tissues (Figure 1F).

The results of the receiver operating characteristic (ROC) analysis results showed the area under the curve (AUC) of TRIM32 in distinguishing normal tissues from CRC tissues was 0.855 (95% CI: 0.808 to 0.901). When the threshold value was set at 0.936, the optimal cutoff point of the model was 5.099. These results imply that high levels of TRIM32 exhibit high accuracy in predicting CRC, and may be a potential diagnostic biomarker for CRC (Figure 1E).

### 2.2. Correlation Analysis Between TRIM32 and the Prognosis of Patients with CRC

To investigate the association between the number of cases with TRIM32 expression and the clinicopathological features of CRC, clinical samples were divided into two groups based on the mean expression level of TRIM32. Samples with expression levels above the mean were defined as high expression. Fisher’s exact test was then used to examine the correlation between the number of cases with high TRIM32 expression and the clinical characteristics of CRC. As shown in Table 1, the expression level of TRIM32 has no significant association with age, sex, clinical T stage, distant metastasis, tumor site, and World Health Organization (WHO) histological classification (all, *p* > 0.05). Fisher’s exact test showed that TRIM32 expression was significantly correlated with lymph node metastasis and tumor TNM stage (both, *p* < 0.05). These results indicate that TRIM32 expression is closely associated with lymph node metastasis and tumor progression in colorectal cancer, suggesting that TRIM32 may play a key regulatory role in colorectal cancer progression and warrants further investigation.

To further clarify the impacts of TRIM32 and other clinicopathological factors on the prognosis of colorectal cancer patients, we conducted a Cox regression analysis. The results of this analysis revealed that factors such as age (≥60 years), lymph node metastasis, distant metastasis, T stage (T3 and T4 stage), TNM stage (stage III and stage IV), and high expression of TRIM32 in tumor tissues were all significantly correlated with the overall survival (OS) of patients (*p* < 0.05). Through in-depth analysis, age, distant metastasis, TNM stage, and TRIM32 expression were identified as independent prognostic factors for OS. Disease-Free Survival (DFS) refers to the survival period without disease. Our research aimed to explore whether TRIM32 would have an impact on DFS. The results indicated that there was no statistical difference between changes in TRIM32 expression and the DFS of colorectal cancer patients (Table 2).

**Figure 2:**
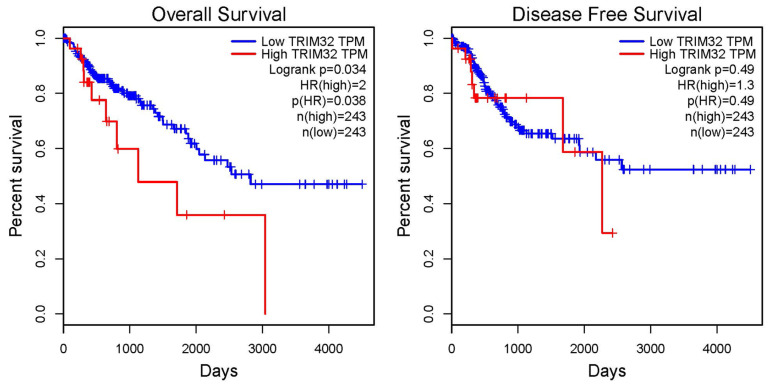
The GEPIA online database was utilized to examine the association between TRIM32 expression level and overall survival (OS) and disease-free survival (DFS) in patients with colorectal cancer.

In order to further explore the relationship between TRIM32 and the prognosis of CRC patients, GEPIA was used to analyze the prognostic significance of TRIM32 in CRC. The *p*-value and hazard ratio (HR) calculated by the log-rank test indicated that high expression of TRIM32 is a risk factor for poor OS of CRC patients (Figure 2, *p* < 0.05).

### 2.3. GO and KEGG Pathway Enrichment Analysis

**Figure 3:**
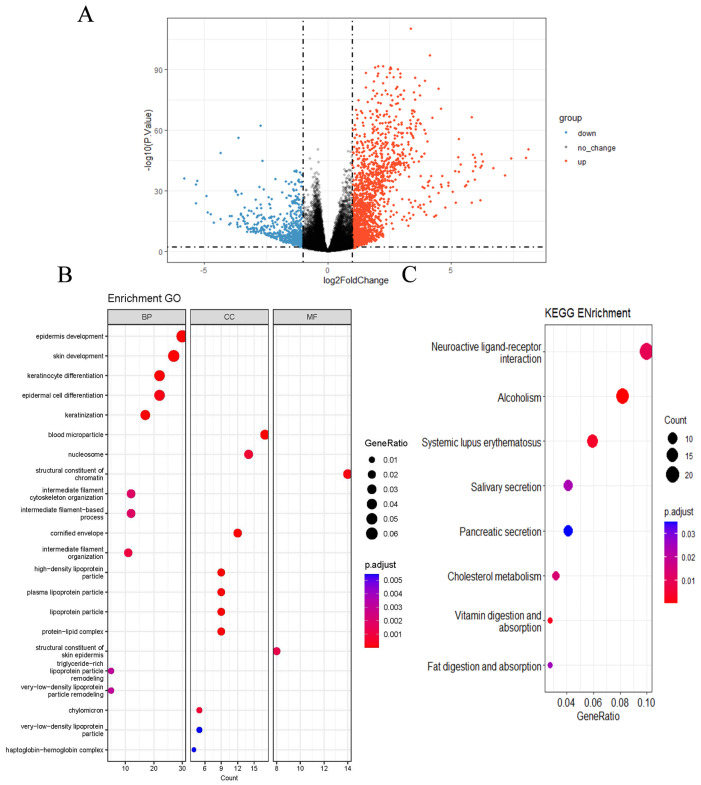
The analysis of the TCGA-COAD/READ dataset and the enrichment analysis of GO and KEGG pathways. (**A**) Volcano plot of differential genes in the TCGA-COAD/READ dataset. (**B**) GO enrichment analysis results of differential genes. (**C**) KEGG Enrichment.

The samples were classified into high- and low-TRIM32 expression groups based on the mean TRIM32 expression level in CRC tissue samples. Differential gene analysis was performed using the limma package, version 4.12.3, and the screening criteria were set as |log2FC| > 1 and *p*.adj < 0.05. A total of 2483 differentially expressed genes (DEGs) related to TRIM32 were obtained, among which 549 were down-regulated and 1934 were up-regulated (Figure 3A). GO enrichment analysis categorizes biological terms into three main categories: molecular function, cellular component, and biological process. This analysis can assist us in identifying key functions related to diseases. In our study, we divided the samples into high- and low-TRIM32 expression groups according to the mean expression level of TRIM32 in colorectal cancer tissue samples. In order to explore the possible biological mechanism of TRIM32 in CRC, GO enrichment revealed that in biological process, TRIM32-related DEGs were mainly involved in the development of the upper epidermis, the differentiation and keratinization of keratinocytes and epidermal cells, the remodeling effect of intermediate filament tissue and intermediate filament cytoskeleton, and triglyceride-rich lipoprotein particles and very low-density lipoprotein particles. In terms of cellular components, TRIM32-related DEGs mainly associated with blood microparticles, nucleosomes, keratinized capsules, high density lipoprotein granules, plasma lipoprotein granules, lipoprotein granules, and other cell compositions. In terms of molecular function, TRIM32-related DEGs were mainly involved in the structural components of chromatin and epidermis of the shin area. Studies have shown that these functions underlie tumor cell differentiation abnormalities, invasion/metastasis, and metabolic reprogramming [23,24], all driving poor CRC prognosis (Figure 3B).

KEGG enrichment analysis indicated that the DEGs related to TRIM32 were mainly enriched in the neuroactive ligand–receptor interaction, alcohol dependence, systemic lupus erythematosus, saliva secretion, pancreatic secretion, cholesterol metabolism, and digestion and absorption pathways of fat and vitamins (Figure 3C).

### 2.4. Verification of shRNA Knockdown Efficiency of TRIM32 in CRC Cells

**Figure 4:**
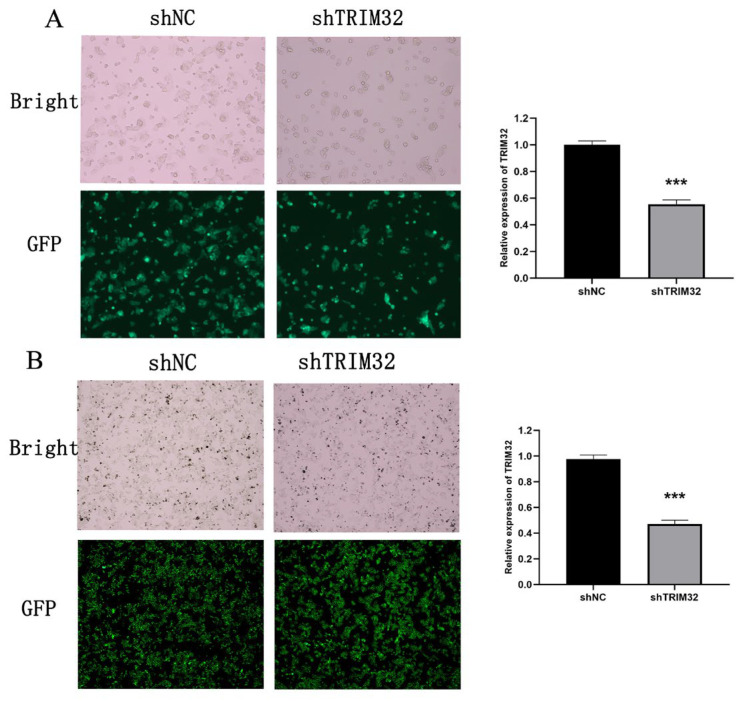
Construction of TRIM32 stable knockdown cell line. (**A**) The morphology and lentiviral infection efficiency (fluorescence microscopy, ×100) of HCT116 cells in shNC and shTRIM32 groups were evaluated, with TRIM32 expression quantified by qPCR. (**B**) The morphology and lentiviral infection efficiency (fluorescence microscopy, ×100) of SW480 cells in shNC and shTRIM32 groups were evaluated, with TRIM32 expression quantified by qPCR (*** *p* < 0.001).

Building on our previous studies [25], we utilized Western blotting to assess the expression levels of TRIM32 in multiple colorectal cancer cell lines, including HT29, SW480, SW620, HCT15, and HCT116. HCT116 and SW480 cells were chosen for lentiviral transfection. The effect of lentivirus infection was monitored under a microscope, and the knockdown efficiency was confirmed by RT-qPCR. The results indicated that the infection efficiency of the lentivirus was approximately 90%. In HCT116 cells, compared with shNC group, the knockdown efficiency in the shTRIM32 group reached 50% (*p* < 0.001) (Figure 4A). In SW480 cells, the knockdown efficiency in the shTRIM32 group was 50%, which was significantly different from that in the shNC group (*p* < 0.001). The Western blot results show that after knocking down TRIM32 in SW480 cells, its expression level is significantly reduced (Figure 4B). Consequently, the shNC and shTRIM32-transfected cells were selected for subsequent cell experiments.

### 2.5. Effect of Knocking Down TRIM32 on the Proliferation, Migration, and Apoptosis of CRC Cells

**Figure 5:**
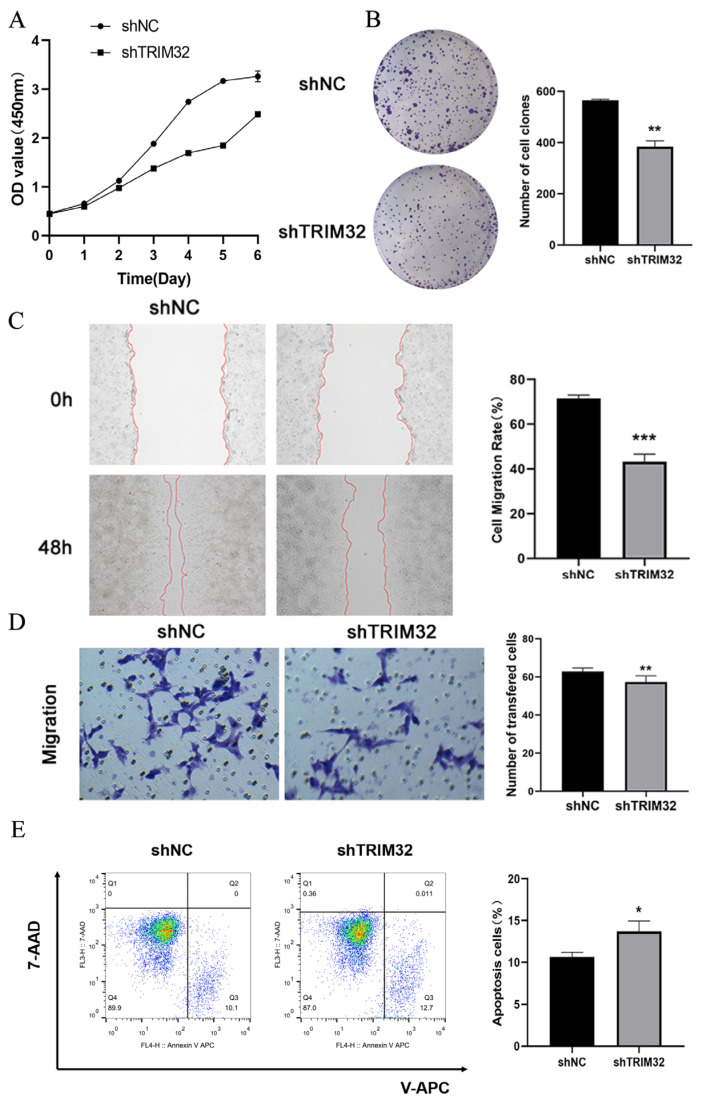
Effect of TRIM32 on the proliferation activity of HCT116 cells. (**A**) The impact of downregulating TRIM32 expression on the proliferation ability of HCT116 cells was assessed using CCK-8 experiments. (**B**) The effect of downregulating TRIM32 expression on the clonogenic ability of HCT116 cells was examined. (**C**) The scratch test was conducted to assess the migratory ability of HCT116 cells following knockdown of TRIM32. The red lines represent the edges of cell migration. (**D**) Transwell migration assay evaluated the impact of TRIM32 on the migratory ability of HCT116 cells. (**E**) TRIM32 induces apoptosis in HCT116 cells (* *p* < 0.05, ** *p* < 0.01, *** *p* < 0.001).

**Figure 6:**
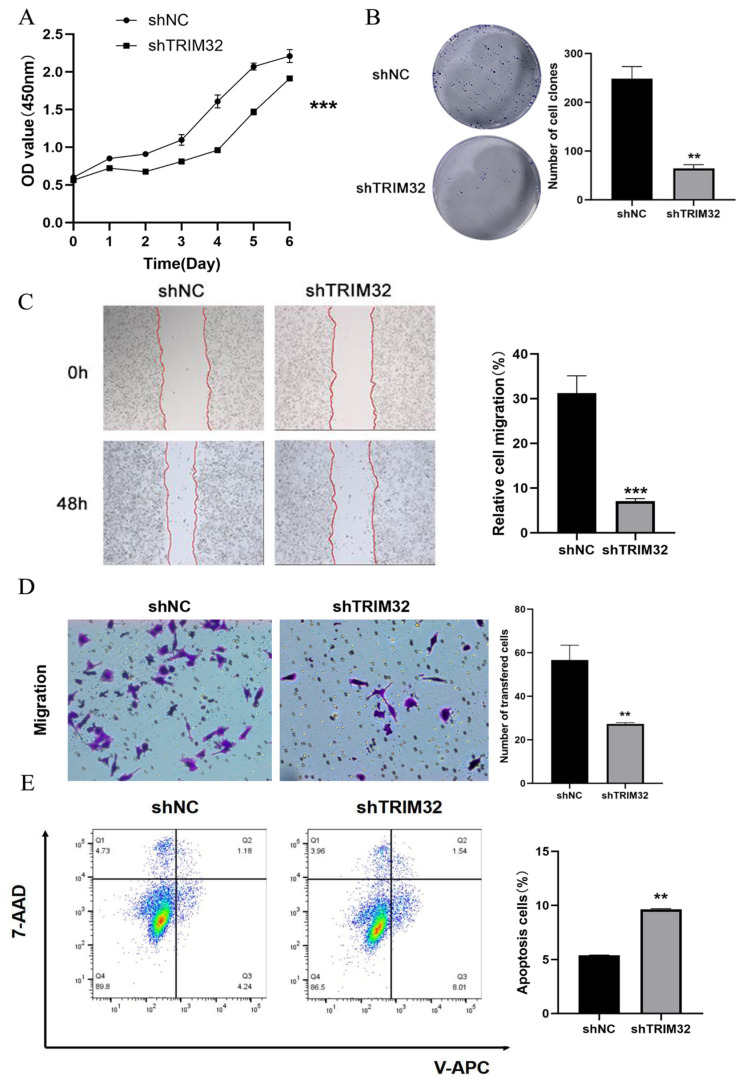
Effect of TRIM32 on the proliferation activity of SW480 cells. (**A**) The impact of downregulating TRIM32 expression on the proliferation ability of SW480 cells was assessed using CCK-8 experiments. (**B**) The effect of downregulating TRIM32 expression on the clonogenic ability of SW480 cells was examined. (**C**) The scratch test was conducted to assess the migratory ability of colorectal cancer SW480 cells following knockdown of TRIM32. The red lines represent the edges of cell migration. (**D**) Transwell migration assay evaluated the impact of TRIM32 on the migratory ability of SW480 cells. (**E**) TRIM32 induces apoptosis in SW480 cells (** *p* < 0.01, *** *p* < 0.001).

In order to evaluate the effect of TRIM32 on the growth and proliferation of HCT116 and SW480 cells, cells transfected with shNC served as the negative control, and the growth curves of cells at 1, 2, 3, 4, 5, 6, and 7 days were drawn. The CCK-8 assay showed that the absorbance of HCT116 and SW480 cells in the shTRIM32 group was significantly lower than of the shNC group (*p* < 0.001, Figure 5A and Figure 6A). The colony formation assay is primarily designed to assess the alterations in cell proliferative capacity. In the present experiment, the impact of TRIM32 on the proliferation of colorectal cancer cells was evaluated by contrasting the quantity, size, and morphological features of the colonies formed by cells in different treatment groups. Two groups were established, including the shNC group and shTRIM32 group. In comparison with the shNC group, the number of clones formed by HCT116 cells in the shTRIM32 group was significantly reduced (*p* < 0.01). Similarly, for SW480 cells, the number of clones in the shTRIM32 group was also notably decreased compared to the shNC group (*p* < 0.01, Figure 5B and Figure 6B). This finding indicates that downregulating the expression of the TRIM32 gene can inhibit the colony-forming ability of both cell lines.

In order to investigate the effect of shTRIM32 on the migration ability of HCT116 and SW480 cells, shNC was used as a negative control, and the healing of cells in the shNC group and shTRIM32 group was recorded 48 h after the scratch in the scratch assay. The results showed that the wound-healing rate of cell scratches in the shTRIM32 group was significantly reduced as compared to shNC group (*p* < 0.01). For SW480 cells, the wound-healing rate in the shTRIM32 group was also significantly lower than that in the shNC group (*p* < 0.01, Figure 5C and Figure 6C).

The effect of shTRIM32 on the migration ability of CRC cells was tested by Transwell assay. The results showed that the number of invasive cells in the shTRIM32 group was significantly lower as compared to the shNC group (*p* < 0.01, Figure 5D and Figure 6D). Thus, TRIM32 can significantly inhibit the cell migration ability of CRC cells.

The effect of TRIM32 on apoptosis of HCT116 and SW480 cells was explored by flow cytometry. The results showed that compared with the shNC group, the proportion of early apoptotic cells and the total proportion of apoptotic cells in the shTRIM32 group increased significantly. After knocking down TRIM32, the total proportion of apoptotic cells in the shNC group of both HCT116 and SW480 cells increased significantly (*p* < 0.05, Figure 5E and Figure 6E).

### 2.6. Knockdown of TRIM32 Inhibits the Growth of CRC Cells by Regulating the NF-κB Signaling Pathway

**Figure 7:**
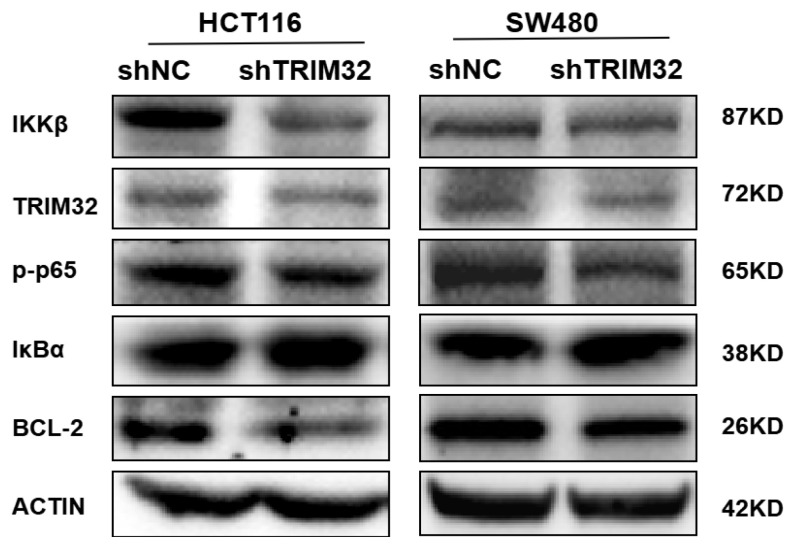
Detection of TRIM32 and relevant NF-κB signaling pathway protein levels by Western blot in HCT116 and SW480 cells.

After CRC cells were infected with TRIM32 shRNA lentivirus, the protein expression levels of NF-κB signaling pathway-related proteins were analyzed by Western blotting. The results showed that compared with shNC group, knocking down TRIM32 in HCT116 and SW480 cells increased IκBα expression and decreased Bcl-2, IKKβ, and p-p65 expression (Figure 7). This indicates that knocking down TRIM32 can inhibit the growth of CRC cells by modulating the NF-κB signaling pathway.

To investigate the regulatory role of TRIM32 in the NF-κB signaling pathway, we conducted relevant experiments. The experimental results showed that, compared with the control group, the protein band intensities of TRIM32 were weakened in the experimental group with knocked-down TRIM32 expression (Figure 7). This directly indicates that knocking down TRIM32 inhibits the activation of the NF-κB signaling pathway.

### 2.7. TRIM32 Knockdown Inhibits the Proliferation of CRC Cells In Vitro

**Figure 8:**
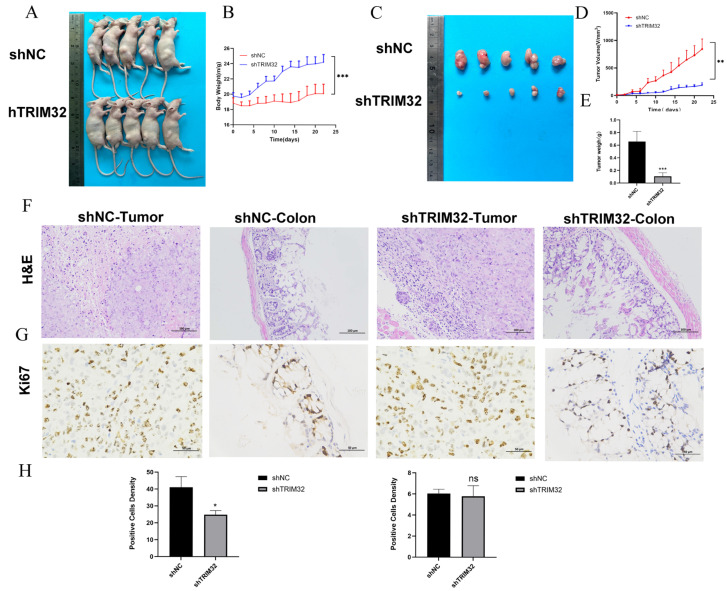
TRIM32 knockdown inhibits CRC cell proliferation in vivo. (**A**) Xenograft gross photographs of BCLB/c nude mice. (**B**) Body weight changes in BCLB/c nude mice. (**C**) Xenograft tumor photographs in BCLB/c nude mice. (**D**) Tumor volume changes in xenografted nude mice. (**E**) Histogram of tumor weight. (**F**) H&E staining results of xenograft tumor and colon tissues in nude mice (×200). (**G**) Ki67 staining of xenograft tumor and colon tissues in nude mice (×400). (**H**) Analysis of Ki67-positive cell rate in xenograft tumor tissues and colon tissues of nude mice (* *p* < 0.05, ** *p* < 0.01, *** *p* < 0.001).

In order to further investigate the role of TRIM32 in tumor growth, experiments were carried out with a HCT116 cell murine subcutaneous xenograft tumor model (Figure 8A). The results showed that the tumors in the shTRIM32 group were significantly smaller than in the control group, which verified that decreased TRIM32 expression can inhibit the growth of CRC (Figure 8B). In addition, the mean weight of mice in the shTRIM32 group was higher than that of the control group. Further analysis showed that the weight and volume of xenograft tumors were significantly affected by the change in TRIM32 expression (Figure 8C). Therefore, when the expression of TRIM32 was knocked down, the growth rate of tumors was slowed, and the weight and volume of the tumors were reduced (Figure 8D,E). This further verified the regulatory role of TRIM32 in the process of CRC tumor growth.

H&E staining of colon tissue showed that compared with the shNC group, a substantial number of goblet cells were observed in the shTRIM32 group, and connective tissue hyperplasia could be seen in the lamina propria in the normal control group (Figure 8F). Results of Ki67 staining showed that compared with the shNC group, tumor cell proliferation and colon malignancy in the ShTRIM32 group showed a decreasing trend (Figure 8G,H).

## 3. Discussion

In certain instances, CRC is hereditary, and genome-wide association studies have shown the importance of genetic factors in CRC [26], as well as their relationships with changes in many oncogenes and tumor suppressor genes [27]. The loss or activation of oncogenes and tumor suppressor genes may promote the occurrence and development of CRC. TRIM32 may be a significant proto-oncogene which exerts a regulatory influence on the occurrence and development of various benign and malignant tumors. In this study, we found that TRIM32 is highly expressed in a variety of cancer tissues through pan-cancer analysis. Analyzing the RNAseq data of CRC in TCGA database and matching samples in the GEO dataset allowed us to verify its expression characteristics, and the possible biological mechanisms of TRIM32 in CRC were further analyzed through GO and KEGG enrichment analyses. Studies have verified that high expression of TRIM32 is related to a poor prognosis of acute myeloid leukemia by using TCGA and GEO databases and clinical sample data [28].

The level of TRIM32 is increased in oral squamous cell carcinoma, and promotes disease progression by enhancing the ubiquitination and degradation of FBP2 [29]. By analyzing CRC-related data in TCGA and GEO databases, we discovered that the expression of TRIM32 was significantly up-regulated in CRC tissues, and high TRIM32 expression was significantly related to lymph node metastasis and TNM stage. In addition, age, distant metastasis, TNM stage, and high TRIM32 expression were independent prognostic factors for CRC patients, and high expression of TRIM32 was significantly related to a poor prognosis of CRC. It has been shown that TRIM32 is highly expressed in gastric cancer, hepatocellular carcinoma, and CRC, and high expression is related to a poor prognosis [30,31,32]. The role and mechanism of TRIM32 in CRC progression and metastasis are still largely unknown; however, TRIM32 may be a potential biomarker to effectively diagnose CRC and evaluate the prognosis of CRC patients in the future.

It has been reported that TRIM32 can promote the progression of many cancers. In glioma tissues, TRIM32 is up-regulated, and its knockdown inhibits cell growth by inducing apoptosis through both p53-dependent and p53-independent mechanisms, identifying TRIM32 as a potential anti-tumor target [33]. In addition, TRIM32 is also a proliferation and anti-apoptosis factor. Studies have shown that when TRIM32 is silenced, the proliferation of gastric cancer cells is significantly inhibited and apoptosis is induced, which indicates that TRIM32 is directly involved in the regulation of the AKT pathway in gastric cancer cells. The silencing of TRIM32 can also effectively inhibit the tumorigenicity of gastric cancer cells in vivo, which highlights the potential value of TRIM32 in the treatment of gastric cancer [14].

In this study, we first constructed a stable TRIM32 knockdown cell line and then verified the knockdown of TRIM32 protein expression through Western blot analysis. Next, we conducted cell-function experiments in HCT116 and SW480 cell lines. The results of CCK-8, colony-formation, wound-healing, Transwell, and flow cytometry assays demonstrated that knocking down TRIM32 significantly inhibited the proliferation and migration of CRC cells while promoting apoptosis.

It has been reported that Bcl-2 is a key inhibitor of apoptosis, and regulates autophagy-related cell death by binding and inhibiting 2 pro-apoptosis proteins, Bax and Bak [34]. Overexpression of Bcl-2 is widely considered to contribute to the occurrence and development of tumors, and even enhance the migratory ability of tumor cells [35]. In this study, Western blotting showed that the knocking down of TRIM32 expression significantly decreased the expression of Bcl-2 in CRC cells. This reveals that TRIM32 may be a positive regulator of Bcl-2 expression, and its down-regulation can promote the apoptosis of CRC cells. The existing literature supports the strategy of inducing apoptosis of tumor cells by regulating the expression of Bcl-2. For instance, Yu et al. found that Se-β-lactoglobulin had a significant cytotoxic effect on human gastric cancer MGC-803 cells by inducing caspase-dependent cell apoptosis, increasing the levels of intracellular ROS and Bax, and down-regulating the expression of Bcl-2, triggering the mitochondrial apoptosis pathway mediated by Bax and Bcl-2 [36]. Although some scholars believe that high expression of Bcl-2 may be regarded as a good prognostic factor [37], it may vary with tumor type, stage, and individual differences. In addition, the combination of 5-fluorouracil and bufalin reduced the expression of anti-apoptosis proteins such as Mcl-1, XIAP, and Bcl-2, and increased the level of pro-apoptosis proteins Bax and Bad, thus inducing apoptosis of HCT116 cells [38]. In colorectal cancer research, significant advancements have been made in understanding the role of Bcl-2. Notably, previous investigations have demonstrated that YAP can interact with TEAD to up-regulate the transcription of Bcl-2. This up-regulation subsequently leads to the inhibition of autophagy and promotes the progression of colorectal cancer, thereby firmly establishing Bcl-2 as a crucial factor in the pathogenesis of CRC [39]. Concurrently, another study has identified that RASSF4 is downregulated in CRC. Interestingly, the overexpression of RASSF4 can not only inhibit cell growth and other related processes but also suppress YAP and Bcl-2. Moreover, knocking down YAP abolishes the effect of RASSF4 on Bcl-2, and it has been shown that TEAD4 can bind to the promoter region of Bcl-2. These findings offer novel insights into the complex regulatory mechanisms involving Bcl-2 and present new directions for future research in this area [40].

NF-κB is a key transcription factor family that regulates inflammation and the immune response. Inhibition of NF-κB activation is important in the occurrence and development of tumors. Under normal circumstances, the NF-κB complex is maintained in the cytoplasm in an inactive form by the inhibitor IκB protein. After stimulation, IκB protein is phosphorylated by IκB kinase (IKK) complexes such as IKKα and IKKβ, and the phosphorylated IκB protein can then be degraded by 26S proteasome [41,42,43], releasing NF-κB to be translocated into the nucleus and activate downstream genes [44]. As the phosphorylated form of p65, an important member of the NF-κB family, the expression level of p-p65 is a key indicator for measuring the activation degree of the NF-κB signaling pathway. TRIM32 is highly expressed in CRC, and knocking down TRIM32 can inhibit the activation of the NF-κB signaling pathway. Our results in HCT116 cells showed that knocking down TRIM32 increased IκBα expression and decreased TRIM32, p-p65, and IKKβ expression. This decrease in p-p65 expression further confirms the inhibitory effect on the NF-κB pathway activation. It suggests that TRIM32 can control the phosphorylation and degradation of IκBα by regulating the activity of IKKβ, and thus control the activation of NF-κB. The stability of IκBα is closely related to the activation of NF-κB, and its synthesis and degradation rate are the key parameters that control the whole NF-κB signaling pathway [45].

Previous studies have shown that TRIM67 inhibits the degradation of IκBα by competitively binding β-TrCP with IκBα, thus inhibiting the activation of NF-κB [46]. In contrast, TRIM32 can promote the activation of NF-κB. It can not only activate IKKβ in cooperation with TRIM56 in response to stimulation of cytoplasmic dsDNA [47], but also interact with TRAF2 to promote the polyubiquitination of K63-linked, thus activating the NF-κB signaling pathway [48]. It has been shown that IKKβ is the upstream kinase activator of IκBα [49]. Many pro-inflammatory stimuli required the IKKβ subunit to activate NF-κB, and IKKβ is the key downstream effector of NF-κB signaling [50]. Genetic research has shown that IKKβ plays a vital role in the activation of NF-κB in TLR signal transduction [51]. In summary, we believe that knocking down the expression of TRIM32 can inhibit the activation of the NF-κB signaling pathway, and thus interfere with the development of CRC.

This study has certain limitations. It utilized only two CRC cell lines, potentially affecting the generalizability to other subtypes, and did not incorporate patient-derived xenografts (PDX) or organoid models. The specific mechanism of shTRIM32 in the process of tumor growth was explored through subcutaneous xenotransplantation experiments. Goblet cells are distinctive epithelial cells in the intestine, primarily responsible for secreting mucus and antibacterial proteins. This secretion constructs a crucial chemical barrier that ensures the stability and health of the intestinal environment [52]. However, the subcutaneous xenograft model, although useful in understanding the role of shTRIM32 in tumor growth, has limitations. It is difficult to fully replicate the intestinal microenvironment. The changes in goblet cells may be affected by the artificial subcutaneous environment and cannot accurately reflect the in vivo situation in the intestine. Future studies should adopt better models to further explore the mechanism of shTRIM32 and its relationship with goblet cells. In the intestinal lamina propria of the shNC group, evident connective tissue hyperplasia was observed. As a pathological alteration, connective tissue hyperplasia disrupts the normal tissue structure and also may affect the functional operation of the intestine, resulting in organ structure damage and dysfunction. This abnormal change in connective tissue is often an important sign of an intestinal inflammatory reaction. Secondly, the activation of cancer-associated fibroblasts (CAFs) may also lead to connective tissue hyperplasia. Once CAFs are activated, they can secrete a large amount of extracellular matrix components, such as collagen and fibronectin, which directly result in an increase in connective tissue. Research has demonstrated that the activation and proliferation of CAFs can prompt the massive production of extracellular matrix (ECM) components. The excessive accumulation of components like collagen and fibronectin is highly likely to induce connective tissue hyperplasia [53]; this indicates that CAF activation is an important driving factor for connective tissue changes in the tumor microenvironment. In addition, CAFs exhibit functional heterogeneity [54]. Some subsets can promote tumor growth and tissue remodeling through various means, which involves inducing connective tissue hyperplasia.

## 4. Materials and Methods

NovoScript Plus All-in-one 1st Strand cDNA Synthesis SuperMix (gDNA Purge), NovoStart SYBR qPCR SuperMix Plus (Novoprotein Scientific Inc., Millburn, NJ, USA). Reagents and devices used in this study included fetal bovine serum (FBS; Hangzhou, China), 1% penicillin-streptomycin double-antibody (Gibco, Grand Island, NY, USA), CO_2_ incubator (Thermo Fisher Scientific, Waltham, MA, USA), Annexin V-APC/7-AAD fluorescent double-staining cell apoptosis detection kit (Jiangsu Kaiji Biotechnology Co., Ltd., Yixing, China), cell proliferation and toxicity detection kit (Guangzhou Rarbio Life Science Co., Ltd., Guangzhou, China), total RNA extraction reagent RNAEx ZOL (Guangzhou Saiguo Biotechnology Co., Ltd., Guangzhou, China), Transwell insert (BD Bioscience, SanJose, CA, USA), actin primary antibody, horse radish peroxidase (HRP)-goat anti-mouse secondary antibody and HRP-goat anti-rabbit secondary antibody (Wuhan Xavier Biotechnology Co., Ltd., Wuhan, China), beta actin primary antibody, goat anti-rabbit IgG secondary antibody and peroxidase-conjugated affinipure goat anti-mouse IgG (H  +  L) secondary antibody (Guangzhou Rarbio Life Science Co., Ltd., Guangzhou, China). TRIM32 (Beijing Boao Sen Biotechnology Co., Ltd., Beijing, China). Bcl-2, IKKβ, and IαKβ (Wuhan Sanying Biotechnology Co., Ltd., Wuhan, China), and Transwell cell culture plates (BD, Franklin Lakes, NJ, USA).

### 4.1. Analysis of TCGA and Gene Expression Omnibus (GEO) Database

Original data of CRC patients were downloaded from TCGA and GEO databases to generate a gene expression matrix. The unpaired differences in the data downloaded from TCGA were compared and analyzed, and the paired differences in the data downloaded from GEO were compared and analyzed. The expression data of TRIM32 in TCGA-COAD and TCGA-READ integrated datasets were analyzed using the pROC package(version 1.18.0), and the receiver operating characteristic (ROC) curve was drawn using the ggplot2 package (version 3.4.0). The TNM staging was corrected, and the samples with incomplete clinical data and prognosis information were deleted. Finally, data of 523 patients were retained. The samples were divided into a high-expression group and a low-expression group, and the correlation between TRIM32 expression and CRC was analyzed.

The clinical characteristics of CRC patients were analyzed by univariate and multivariate COX regression analysis. Kaplan–Meier survival analysis was carried out on the integrated clinical data using the Survival package(version 3.5-5)to determine the correlation between TRIM32 expression and the prognosis of patients with CRC. Differential gene analysis was carried out using the limma package, and the screening conditions were set as: |log2FC| > 1 and *p* adjusted (*p*.adj) < 0.05. The differential gene list was analyzed and visualized by Gene Ontology (GO) and Kyoto Encyclopedia of Genes and Genomes (KEGG) enrichment with the clusterProfiler package, org.Hs.eg.db package, and ggplot2 package.

### 4.2. Cell Culture

The human colon cancer cell line HCT116 and SW480 (Gift from the Cancer Research Institute, School of Basic Medicine, Southern Medical University) was used. HCT116 and SW480 cells were cultured in DMEM, supplemented with 10% FBS and 1% penicillin-streptomycin antibody, and incubated in a 5% CO_2_ incubator at 37 °C.

### 4.3. Construction of Stably Transformed Cell Line by Lentivirus Transfection

HEK293T cells (5 × 10^6^) were inoculated into a 10 cm dish, and lentivirus was packaged with DNA solution (GV vector plasmid 20 μg + pHelper1.0 vector plasmid 15 μg + pHelper2.0 vector plasmid 10 μg). The number of cells was adjusted to 5.5 × 10^4^/mL, and placed in 12-well plates. A total of 500 μL of complete medium containing HiTransG P was added to each well, followed by addition of 10 μL of virus solution (titer 1 × 10^8^ TU/mL, MOI = 10). The virus solution and the culture medium were fully mixed, and after about 72 h, the cells were observed using a fluorescence microscope. After the infection efficiency was confirmed, the cells were screened using puromycin, and they were used in the subsequent experiments.

### 4.4. Cell Plate Clone Formation Experiment

Cells in logarithmic growth phase (500 to 1000) were inoculated into a 6-well plate, and culture was stopped when cell colonies were visible to the naked eye. Paraformaldehyde (1 mL, 4%) was added to the wells and the cells were fixed for 1 h. The cells were then stained with 1 mL of 1% crystal violet for 20 min. Photos were taken, and the colonies were counted.

### 4.5. Cell Scratch Test

Five horizontal lines were drawn evenly on the back of 6-well cell culture plates, with one line every 0.5–1 cm. Cells in logarithmic growth phase were inoculated into the 6-well plates (5 × 10^5^ cells/mL). After 24 h of cell growth, the cells were scratched with a sterilized 200 μL pipette gun head perpendicular to the scribe lines. The plates were washed 3 times with PBS to remove the scratched cells. Photographs of the plates were taken using a light microscope at 0, 24, and 48 h. Image J software (version 1.53t) was used to process the data and analyze the results.

### 4.6. Cell Counting Kit (CCK-8) Assay

The HCT116 and SW480 cells were inoculated into 96-well plates (1 × 10^5^ cells/mL), and cultured in a 5% CO_2_ incubator at 37 °C for 48 h. Fresh culture medium (100 μL) and 10 μL of CCK-8 reagent were added to each well and evenly mixed. Incubation was continued for 2 h. The optical density (OD) values at 450 nm of each group were measured by an enzyme-labeled immunosorbent assay reader. The formulas are as follows: cell survival rate = [(As − Ab)/(Ac − Ab)] × 100%, and inhibition rate = [(Ac − As)/(Ac − Ab)] × 100%.

### 4.7. Transwell MIGRATION Experiment

The Transwell migration assay was performed using a Transwell insert with an 8 μm pore size filter. A total of 1 × 10^5^ cells from each group in a logarithmic growth period were obtained and added to 100 μL of serum-free medium. After resuspending the cells, the cell suspension was added to the upper chamber of a Transwell cell culture plate, and 600 μL complete medium was added to the lower chamber. After incubation for 12 to 48 h, the chamber was taken out and the cells in the upper chamber were wiped off with a cotton swab; 4% paraformaldehyde was added for fixation; 0.1% crystal violet was added for dyeing for 10 min, and then the chamber was washed 5 times with PBS. After drying, photos were captured under a light microscope for analysis.

### 4.8. Apoptosis Assay

A total of 0.5 mL of HCT116 and SW480 cell suspension (5 × 10^5^ cells) was obtained and transferred to a clean 1.5 mL centrifuge tube. Next, 1.25 μL of Annexin V-APC was added and the reaction was allowed to proceed for 15 min in the dark at room temperature (18–24 °C). The cells were centrifuged at 1000× *g* for 5 min at room temperature, and then the supernatant was removed. The cells were resuspended in 0.5 mL of precooled PBS buffer, and 10 μL of 7-AAD was added and mixed well. The samples were stored on ice in the dark, and the incubated cells were detected and analyzed by flow cytometry. The flow cytometer we used is BD Calibur, the total number of counted events is 5 × 10^5^.

### 4.9. Detection of mRNA Expression by Fluorescence Quantitative PCR

Total RNA was extracted with TRIzol reagent, and a Novoprotein reverse transcription kit was used to complete the synthesis of cDNA. The qPCR reaction was carried out with the 20 μL reaction system of 2 × NovoStar SYBR qPCR SuperMix of Novoprotein. Two pairs of primers with high specificity were designed and synthesized using Primer Premier software (version 6.24), with the following sequences: TRIM32 forward: 5′-CCGGGAAGTGCTAGAATGCC-3′, reverse: 5′-CAGCGGACACCATTGATGCT-3′; GAPDH forward: 5′-GTCTCCTCTGACTTCAACAGCG-3′, and reverse: 5′-ACCACCCTGTTGCTGTAGCCAA-3′. The sequence was synthesized by Shanghai Shenggong Bioengineering Technology Service Company. The Sensoquest LabCycler gradient PCR instrument is sourced from SensoQuest GmbH, Göttingen, Germany. The LightCycler^®^ 96 real-time fluorescent quantitative PCR instrument is sourced from Roche, Switzerland.

### 4.10. Detection of Related Proteins by Western Blotting

After cell culture, the mixture of lysate and protease inhibitor was lysed for 10 min, and then was centrifuged for 10 min at 12,000 rpm at 4 °C. The supernatant was collected, and the protein concentration was determined by the Bradford method. The same amount of protein was electrophoresed by SDS-PAGE, and transferred to a PVDF membrane that was cut in advance and was consistent with the size of the separation gel; 5% milk was added for sealing. The diluted primary antibody was added, and incubation was performed overnight at 4 °C. The diluted secondary antibody was added, and the mixture was incubated for 30 min. After incubation, TBST was added and eluted on a shaker for 5 min. Enhanced chemiluminescence (ECL) was used to detect protein bands. Exposure and image collection were performed with an ECL luminometer.

### 4.11. Xenotransplantation Model

Four-week-old male BALB/c nude mice were purchased from Guangdong Medical Laboratory Animal Center, and after 3 days of adaptive feeding, a model was established. The mice were randomly divided into an shNC group and an shTRIM32 group, with 5 mice in each group. The shNC and shTRIM32 cells, transfected from HCT116 cells, were in the logarithmic growth phase. The cell density of each group was adjusted to 1 × 10^7^ cells/mL, and 0.1 mL of tumor cells was inoculated subcutaneously in the right hind limb of each mouse. Tumor growth was observed every day, the long diameter (L) and short diameter (W) of the tumors were measured, and tumor volume was calculated with the formula: tumor volume = 1/2 L × W^2^. The weight of the mice was also recorded daily. On the 21st day, the nude mice were euthanized using the carbon dioxide asphyxiation method, the subcutaneous tumor tissues were removed and tumor weight and size were recorded.

### 4.12. H&E Staining

After the mice were killed, the colon and tumor sections were washed with PBS, then immediately fixed in 10% formalin, dehydrated, and embedded in paraffin. The paraffin blocks were cut into 4 μm sections using a microtome (RM2016, Leica Microsystems Shanghai Co., Ltd., Shanghai, China). Subsequently, the sections were stained with hematoxylin dye solution and eosin and then sealed with neutral gum. Microscopic examination was carried out using a Nikon Eclipse E100 upright optical microscope (Nikon, Tokyo, Japan), and images were captured.

### 4.13. Ki67 Immunohistochemistry

The paraffin sections were dewaxed by immersion in the environmental-friendly dewaxing solution I, II, and III for 10 min each, followed by treatment with absolute ethanol I, II, and III, and then rinsed with distilled water. Subsequently, antigen retrieval was performed using 20 × Tris-EDTA (pH 8.0) antigen retrieval buffer by heating for 30 min. After cooling, the sections were washed with PBS. Next, endogenous peroxidase was blocked by incubating the sections in 3% hydrogen peroxide solution. After washing with PBS, 3% BSA was dropped onto the sections and incubated at room temperature for 30 min for blocking. The blocking solution was gently shaken off, and the primary antibody Ki67 (diluted 1:500) was added. The sections were then incubated overnight in a humid chamber at 4 °C. After incubation, the sections were washed with PBS, and the HRP-labeled goat anti-rabbit IgG secondary antibody was added and incubated at room temperature for 50 min. After another washing, the DAB chromogenic solution was added for staining. The reaction was terminated by rinsing with tap water. Then, the sections underwent hematoxylin counterstaining, differentiation, blue-returning, and dehydration and mounting steps. Finally, the results were interpreted using the E100 white light microscope manufactured by Nikon Instruments Co., Ltd., Tokyo, Japan.

### 4.14. Statistical Analysis

R Studio (version 4.2.2) was used for bioinformatics analysis. The paired *t*-test was used to analyze the expression differences in TRIM32 in paired samples of the GEO dataset, and the independent sample *t*-test was used to analyze the expression differences in TRIM32 in TCGA-COAD/RESD downloaded from TCGA database, and Fisher’s exact test was used to analyze the correlation between TRIM32 expression and clinical factors related to CRC prognosis. The Kaplan–Meier method was used for survival analysis, and univariate and multivariate COX regression were used to analyze the influence of prognostic factors on survival. GraphPad Prism 8.0 and IBM SPSS version 26.0 were used for statistical analysis and drawing statistical charts. An independent sample *t*-test or one-way ANOVA was used to analyze data with a normal distribution, and a nonparametric test was used to analyze data without a normal distribution. A homogeneity test of variance was used for statistical analysis; all analyses were 2-tailed, and a value of *p* < 0.05 was considered to indicate a statistically significant difference.

## 5. Conclusions

The results of this study showed that the expression of TRIM32 in CRC tissue is significantly up-regulated, and high TRIM32 is associated with poor OS of patients with CRC. Age, distant metastasis, tumor TNM stage, and TRIM32 expression are independent prognostic factors for OS. When TRIM32 expression is down-regulated, it can significantly inhibit cell proliferation and clonal formation. In addition, our results also showed that knocking down TRIM32 increased the expression of IκBα and decreased the expression of TRIM32, p-p65, Bcl-2, and IKKβ. This suggested that TRIM32 can regulate the phosphorylation and degradation of IκBα by regulating the activity of IKKβ, thereby leading to the observed decrease in p-p65 expression and ultimately controlling the activation of NF-κB. We also established a mouse model of ectopic tumors to further confirm that downregulation of TRIM32 can significantly inhibit the proliferation of CRC cells in vivo. These findings indicate that the knockdown of TRIM32 effectively inhibits the activation of the NF-κB signaling pathway. Taken together, knockdown of TRIM32 can effectively curb the development of CRC; thus, TRIM32 may become a potential target in the treatment of CRC.

## Figures and Tables

**Table 1:** The relationship between the number of cases with high TRIM32 expression levels and clinical characteristics in the TCGA colorectal cancer database (* *p* < 0.05).

Clinical Case Factors		Case Number	High Expression Cases (%)	*p*-Value
Age	<60	147	57 (38.8)	0.55
	≥60	376	158 (42.0)	
Gender	male	276	122 (44.2)	0.13
	female	247	93 (37.6)	
T stage	T1 + T2	103	45 (43.6)	0.58
	T3 + T4	420	170 (40.5)	
Lymph node metastasis	no	308	138 (44.8)	0.047 *
	yes	215	77 (35.8)	
Distant metastasis	MX	48	15 (31.3)	0.34
	no	398	168 (42.2)	
	yes	77	32 (41.6)	
TNM staging of tumor	I + II	299	135 (45.2)	0.031 *
	III + IV	224	80 (35.7)	
Tumor site	ascending colon	83	31 (37.3)	0.99
	cecum	87	35 (40.2)	
	colon	108	47 (43.5)	
	descending colon	17	6 (35.3)	
	hepatic flexure of the colon	12	5 (41.7)	
	rectosigmoid junction	5	2 (40.0)	
	rectum	83	37 (44.6)	
	sigmoid colon	109	44 (40.4)	
	transverse colon	19	8 (42.1)	
WHO pathological type	tubulovillous adenoma	7	2 (28.6)	0.17
	in adenocarcinoma			
	mixed subtype adenocarcinoma	3	2 (66.7)	
	adenocarcinoma NOS	435	170 (39.1)	
	adenosquamous carcinoma	1	1 (100)	
	carcinoma NOS	1	1 (100)	
	mucinous adenocarcinoma	68	36 (52.3)	
	papillary adenocarcinoma NOS	2	0 (0)	
	tubular adenocarcinoma	5	3 (100)	
	adenocarcinoma with	1	0 (0)	
	neuroendocrine differentiation			

**Table 2:** Univariate COX regression analysis and multivariate COX regression analysis of clinical characteristics of colorectal cancer patients (* *p* < 0.05, ** *p* < 0.01, *** *p* < 0.001).

Fator	Univariate COX Regression Analysis		Multivariate COX Regression Analysis	
	HR (95%CI)	*p*-Value	HR (95%CI)	*p*-Value
Age(<60 years vs. ≥60 years)	1.70 (1.00–2.70)	<0.05 *	2.25 (1.34–3.77)	<0.01 **
Gender(Male vs. Female)	0.93 (0.62–1.40)	>0.05		
Lymph node metastasis (absent vs. present)	2.60 (1.70–4.00)	<0.001 ***	0.53 (0.18–1.54)	>0.05
Distant metastasis (absent vs. present)	4.60 (3.00–7.0)	<0.001 ***	2.96 (1.78–4.93)	<0.001 ***
T stage (T1 + T2 vs. T3 + T4)	2.90 (1.30–6.20)	<0.01 **	1.84 (0.83–4.09)	>0.05
TNM tumor stage (stage I + II vs. stage III + IV)	3.00 (2.00–4.60)	<0.001 ***	2.98 (1.79–4.96)	<0.05 *
TRIM32 (low expression vs. high expression)	1.50 (1.00–2.30)	<0.05 *	1.57 (1.05–2.34)	<0.05 *

## Data Availability

All data are included within the main text. Additional data that support the findings in this study are available from the corresponding author upon reasonable request.
